# Characterization of Joints between Carbon Fiber Composite Parts Using a Microstrip Transmission Line Method

**DOI:** 10.3390/s21041142

**Published:** 2021-02-06

**Authors:** Borja Plaza, Daniel López, David Poyatos

**Affiliations:** 1 Radiofrequency Area, National Institute for Aerospace Technology (INTA), Torrejon de Ardoz, 28850 Madrid, Spain; plazagb@inta.es; 2 Electromagnetic Compatibility Area, National Institute for Aerospace Technology (INTA), Torrejon de Ardoz, 28850 Madrid, Spain; lopezsd@inta.es

**Keywords:** carbon fiber composite, electromagnetic characterization, electromagnetic environmental effects, lightning, microstrip transmission line

## Abstract

The electromagnetic performance of aerial platforms, which are composed mostly of nonmetallic materials, is a subject of great interest at present time. The behavior of this type of composite structure against electromagnetic environmental effects (E3), such as lightning, is not well-studied as in the case of metalic structures. The purpose of this article is to characterize the joints present in aerial platforms constructed mainly of nonmetallic composite materials. The study of these joints is fundamental because electrical discontinuities or preferential routes can produce changes in the electromagnetic behavior of an aircraft. The proposed measurement system for the characterization of these joints is a microstrip line. The flexibility of the test setup allows for evaluation of different joints in carbon fiber composite (CFC) samples with a different number of plies. Additionally, approximated models of the behavior of the joints as well as the detection of possible defects in the joining process are reported.

## 1. Introduction

In recent years, the use of nonmetallic composite materials for the design and manufacture of aeronautical platforms has become widespread [1]. Specifically, the utilization of carbon fiber composite (CFC) has increased in aircraft structures because of their high strength and high stiffness per density compared with conventional metallic materials [2]. One of the drawbacks of the employment of these composite materials is that their behavior against electromagnetic environmental effects (E3) is less known [3].

The aim of the UAVE3 project [4] and its continuation, the eSAFE-UAV project, is the study of these effects in unmanned aerial vehicles (UAV), where it is not rare that the main constituent material is CFC. The work presented in this paper is part of these projects, particularly in the context of the effects of lightning strikes on the structure of an UAV manufactured mostly of CFC.

The currents of a lightning strike and the associated electromagnetic fields generated by them could penetrate inside any aerial platform and could produce disturbances in the electronics and processors that make up the avionics systems. This threat is bigger in the case of vehicles with composite material structures [5,6]. The absence of uniformity in the conduction of electricity in the different directions makes the study of lightning effects on CFC structures especially important [7].

Just as the studies on the electromagnetic characterization of materials [8,9,10,11] and, in particular, on the electrical conductivity of CFC composites [12,13,14,15,16] have been multiplied, knowledge on the electromagnetic performance of the union of parts made of CFC structures is not as far developed compared to parts made of metal [17]. The study of these joints is fundamental because electrical discontinuities or preferential paths can produce changes in the aircraft electromagnetic behavior.

Three main processes are used to join structural aeronautical parts: fastener joining, adhesive bonding, and welding [18]. The first two applications are used for CFC structures. Welding is applied in metallic structures; however, it can also be used to join ceramic and thermoplastic polymer components.

Fastener joining is widely used [19] due to a number of advantages such as a low-cost process; no special joint preparation; and the possibility, depending on the type of fastener employed, to disassemble and replace the parts. The application of adhesives in primary structures has been found in metallic and nonmetallic joints, as in the case of the union of stringers to the skin in the fuselage and wings [20]. Another example can be found in the Boeing 787 Dreamliner, partially bonded with adhesives. This includes the outside covering (or nacelle) of the jet engine, the wing flaps, interior luggage spaces, bathrooms, and floors [21].

**Figure 1:**
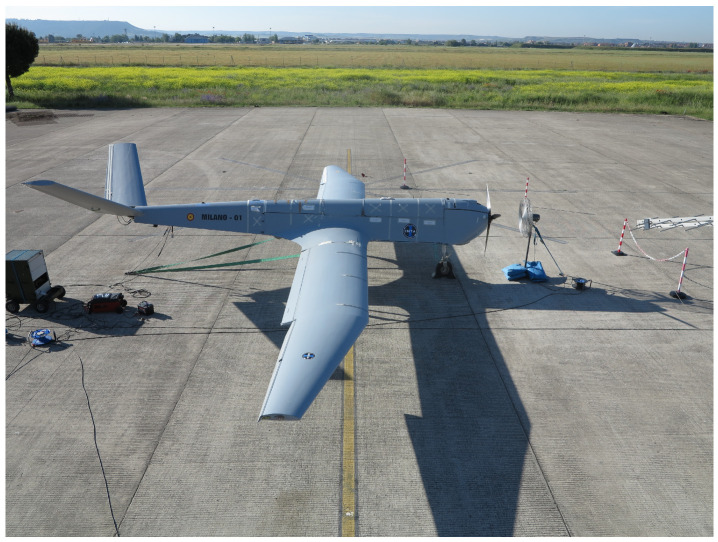
MILANO: a medium altitude long endurance remotely piloted aircraft system development at the National Institute for Aerospace Technology (INTA).

In this study, joints from an actual aerial vehicle, the MILANO (Figure 1), will be used as an example. The MILANO [22] is a medium altitude long endurance (MALE) remotely piloted aircraft system (RPAS) under development at the National Institute for Aerospace Technology (INTA). It constitutes a real case of RPAS that is quite representative of current aerial platforms trends.

Studies of joints in CFC structures are scarce in the bibliography and limited to electric resistance methods [23,24,25], dedicated to monitor the potential drop across the sample under test. These methods are effective at frequencies up to 1 MHz. For higher frequencies, they start to fail [26]. As the lightning effect ranges from a few Hz to 10 MHz (see Section 3.2), new methods must be researched in order to assess the behavior of joints up to this frequency.

The measurement system proposed in this work for the characterization of these joints at these frequencies is based on a microstrip line. This type of transmission line has been used in test methods for many applications such as the characterization of magnetic composite materials [27] or the determination of the permittivity of dielectric substrates [28] in addition to the damage detection of CFC composites [29].

In this paper, the proposed method implies the substitution of an upper conductor of the microstrip line for several samples of CFC parts joined by rivets or adhesives. Under these circumstances, in a first measurement, the scattering parameters (S-parameters) of the line are retrieved. The S-parameters describe the electrical behavior of linear electrical networks when undergoing various steady state stimuli by electrical signals. Representation in accordance with the ideas of incident, reflected, and transmitted waves is given by a scattering matrix [30].

In a second measurement, a surface current probe is also employed to obtain the current that flows through the joint under analysis. The samples are fabricated after the assessment of typical joints and CFC laminates of the MILANO.

The article is organized as follows: the samples used during the tests are defined in Section 2. Both the materials and joints of the samples are described in this section. Section 3 introduces the measurement method and the tests to be carried out. The combined measurement of the S-parameters and the surface current will allow us to obtain information about the behavior of the joints. The results obtained, together with a proper interpretation of them, are shown in Section 4. Finally, Section 5 draws the conclusions of the study.

## 2. Test Samples

The MILANO RPAS has a wingspan of 12.5 m, a length of 8.52 m, and a height of 1.43 m and is practically entirely made of CFC. The central fuselage was reviewed for the most typical, repeated, and representative laminates and joints of the structure.

### 2.1. Materials

In this study, four laminates of the central fuselage of the MILANO were selected and specimens were fabricated out of carbon-epoxy prepreg tapes. The epoxy matrix used was Cytec MTM^®^45-1 with HexTow^®^ IM7 carbon fiber based on unidirectional style for each ply. The name given to each laminate, the particular number of plies, and the stacking sequence are summarized in Table 1. The thickness of the cured ply was nominally 128 μm; therefore, the thickness of the samples evaluated was between 1.536 mm and 4.608 mm, depending on the number of plies described in Table 1.

### 2.2. Joints

The examined part of the MILANO used mainly three types of joints. The first joint included an adhesive bonding process, and the other two were based on a fastener joining process. In order to characterize these kinds of joints, ad hoc test samples were fabricated. They were composed of two CFC parts joined by one of the three possible options. The samples all had the same dimensions: 396 mm length and 260 mm width, while the joining area had a length of 260 mm and a width of 56 mm.

**Figure 2:**
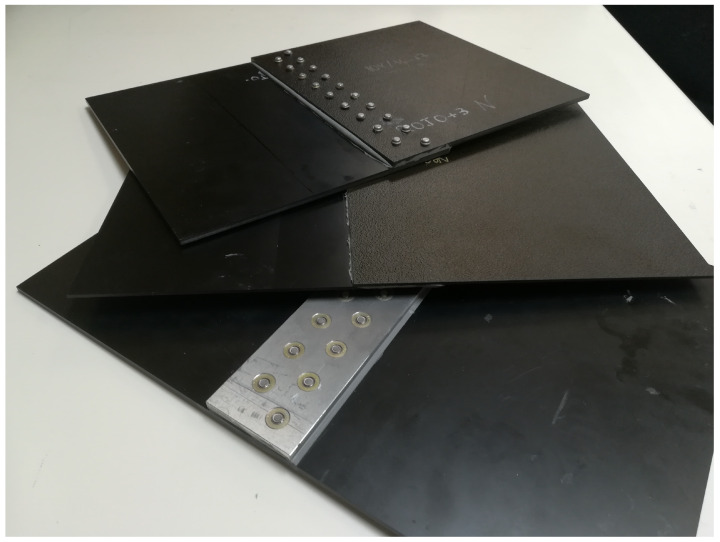
Carbon fiber composite (CFC) joints of the MILANO remotely piloted aircraft system (RPAS) structure. Top: material–rivets–material; middle: material–material; and bottom: material–aluminum–material.

The samples were codenamed for this study as follows: material–material for adhesive joints (blue–blue is an adhesive joint between two parts of the stack material blue in Table 1); material–rivets–material for joints where the element that directly connects the two materials are rivets (example red+3–rivets–red+3); and material–aluminum–material for joints where each of the CFC parts is attached by rivets to an aluminum plate (example red+3–aluminum–red+3). The last two joints were only made with red+3 material (see Table 1) because a minimum thickness of the sample was needed for the rivets. Note that, for material–material and material–rivets–material, the CFCs overlap while, for material–aluminum–material, only the aluminum plate overlaps. The CFC joints are shown in Figure 2.

For the adhesive joints, Hysol^®^ EA9394 based on epoxy resins with cold curing was used. The adhesive was applied on the overlapping area between the two joined panels. As described before, this area had a length of 260 mm and a width of 56 mm.

On the other hand, for both fastener joints, Cherry Titanium Maxibolt^®^ rivets, FSCM 11,815 type, were used. These types of blind head rivets are the most commonly used in aerospace applications in order to not deteriorate the aerodynamics of the aircraft by hiding the rivets into the fuselage. The position of the rivets within the sample was the same for both types of fastener joints. The distance between rows of rivets was 24 mm and between rivets in the same row was 32 mm. For the material–rivets–material joints, the overlapping area had a width of 56 mm while, for the material–aluminum–material joints, the aluminum plate (aluminum 7075-T6 [31]) had a length of 260 mm, a width of 56 mm, and a thickness of 5 mm (see Figure 2).

## 3. Measurement Methods

### 3.1. Test Setup

**Figure 3:**
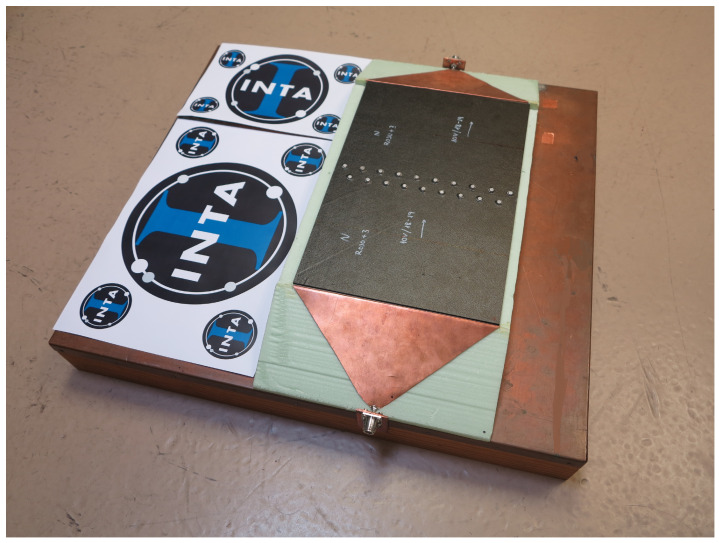
Microstrip line configuration for characterize the joints present in the MILANO RPAS.

In this paper, a microstrip line configuration was proposed to characterize the joints presented in a CFC manufactured aircraft. The upper conductor of this transmission line was substituted by the 396 × 260 mm samples under test as just described (Figure 3). The parameters of the proposed microstrip line are detailed next.

The measurement instrument was a Rhode-Schwarz^®^ ZNC vector network analyzer (VNA) (frequency range from 9 kHz to 3 GHz). Type N connectors were placed on both sides of the microstrip line and connected with the VNA using 50 ohm coaxial cables. The strip were wide enough to accommodate samples with joints and, at the same time, to comply with the 50 ohm impedance of the connectors, the cables, and the VNA. Thus, a characteristic impedance of 50 ohm was chosen also for the microstrip line. Taking into account the material sample width of 260 mm and the availability of a 50 mm high block of Styrodur^®^ ($\epsilon_{r_{Styrodur}}\approx$ 1) [32], a transmission line impedance close enough to 50 ohm was achieved [33].

**Figure 4:**
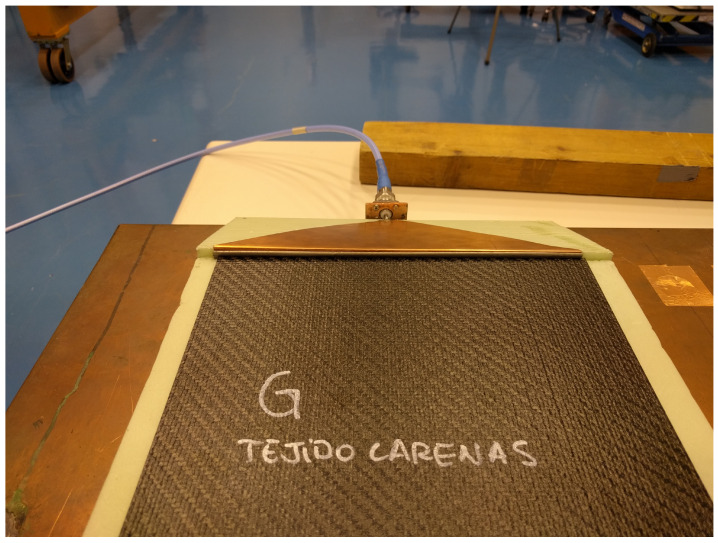
Metallic gasket to achieve a good connection between the triangular transition and the sample.

The height of the substrate implied the design of a transition from the inner conductor of the type N connector to the strip of the microstrip line. This was done through a 50 ohm triangular transition that offered low return losses. With this triangular transition, a uniform injection throughout the sample was obtained. To achieve a good connection between the triangular transition and the sample, a metal tab with a metallic gasket was used as shown in Figure 4.

### 3.2. Measurements

The operating frequency band for the microstrip line test setup was selected according to the frequency range that represents the transients generated by a direct lightning strike and the subsequent indirect effects.

According to aeronautical regulations [34], the main components of a direct lightning strike include a rise time between 1 μs and 250 μs and a pulse duration that reach up to milliseconds, which means current and voltage waveforms for which the frequency range is within a few Hz and 1 MHz, approximately.

Also, the energy from a lightning strike couples through different mechanisms to electronic systems and materials. These lightning indirect effects (LIE) produce damped sine waveforms up to 10 MHz of fundamental frequency and double exponential pulses with rise times up to 0.1 μs and time duration within 10 μs and several hundreds of microseconds. That type of pulse implies frequency ranges from a few kHz to 10 MHz. The waveform of the pulses can be checked in RTCA/DO-160D [35].

For all of the above, it can be estimated that a correct frequency band for analyzing the behavior of materials and joints in a lightning strike environment can be set from a few Hz to 10 MHz.

As explained next, measurements of the S-parameters and surface currents were carried out. In both cases, the upper conductor of the proposed microstrip line was replaced with the samples under test and the measurements were simply made with the VNA. The operation conditions of the VNA were: frequency range between 9 kHz and 1 GHz (3001 points), measurement bandwidth of 700 Hz, and source power generating 10 dBm. The tests were conducted at the premises of the National Institute for Aerospace Technology (INTA), Spain, by accredited expert members of the electromagnetic compatibility area.

#### 3.2.1. Scattering Parameters Measurements

For the first type of measurement, the S-parameters of the line were retrieved with the VNA between 9 kHz and 10 MHz. The selection of this frequency band was based on the typical LIE frequency band aforementioned.

#### 3.2.2. Surface Current Measurements

**Figure 5:**
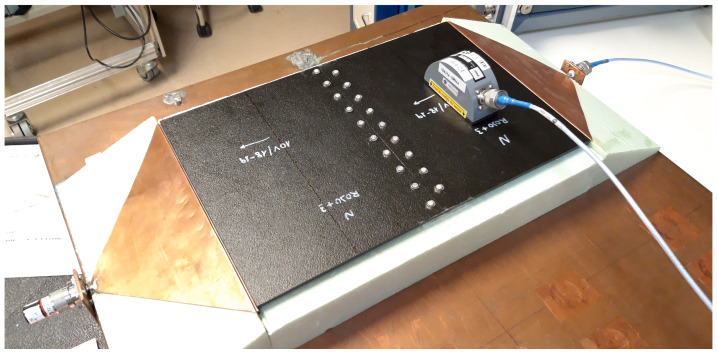
Surface current measurement with a multi-gap loop B-dot ground plane sensor.

In this second type of measurement, a multi-gap loop B-dot ground plane sensor F-90 of Fischer Custom Communications, Inc. [36] was used to retrieve the current on the surface of the samples under test, and the results obtained are within the frequency range of 9 kHz to 1 MHz (see Figure 5). The measurement of surface currents was limited to 1 MHz because the transients of a direct lightning strike are within that band.

**Figure 6:**
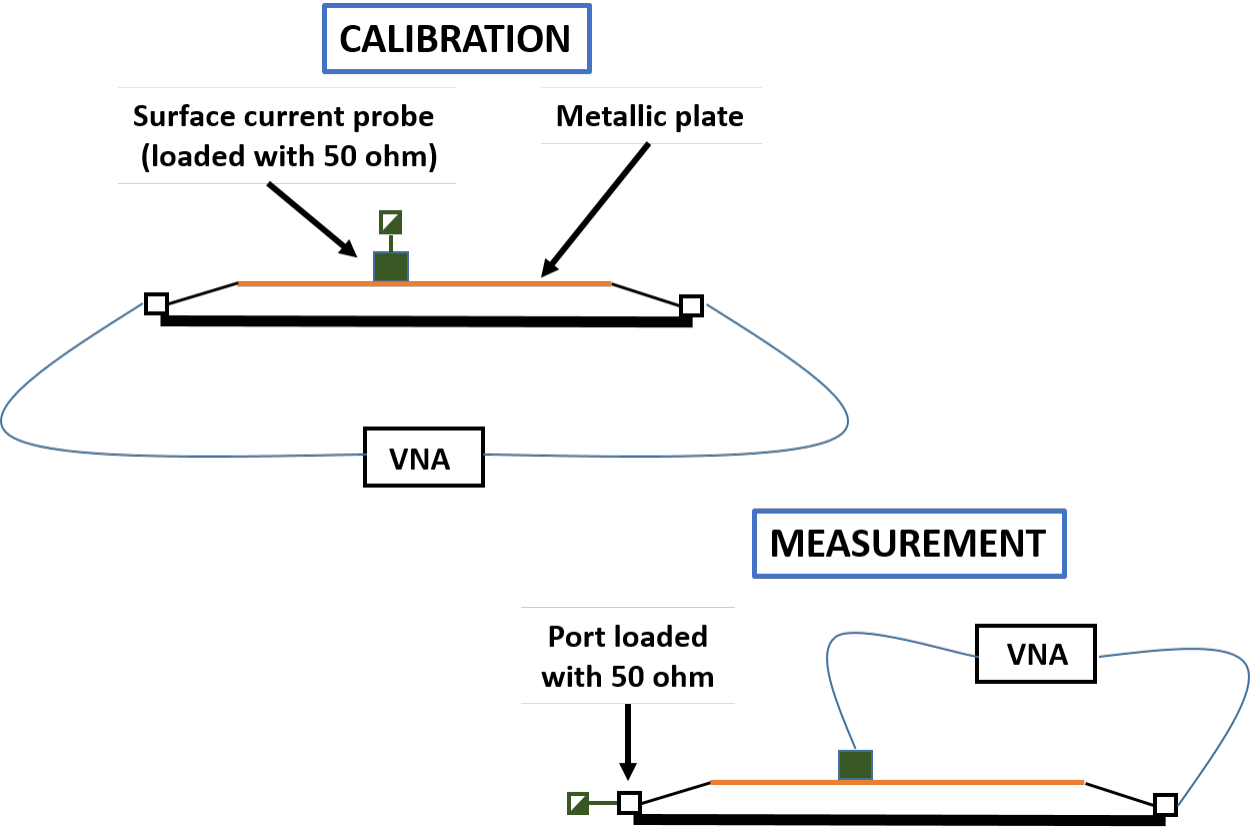
Surface current measurement and calibration with a multi-gap loop B-dot ground plane sensor on the samples surface under test.

The surface current measurements presented here are relative measurements with respect to a calibrated reference level. The aim of this calibration is to take into account the effect of the proposed setup, including the multi-gap sensor, and to remove it from the measurements in order to obtain only the contribution of the joint. Only the effect of the joint is relevant; therefore, the current that flows through the microstrip when the calibration plate is included and the probe is loaded was measured during calibration to be subtracted from the measured current when the joint is present. Figure 6 explains the procedure. For calibration, a metallic plate made of homogeneous 7075-T6 aluminium [31] with a thickness of 5 mm and the same dimensions of the sample under test substituted the upper conductor of the microstrip line, and the N connectors of the line were connected to the VNA while the sensor was charged with a 50 ohm load termination. The $S_{21}$ parameter was thus measured and stored. After that, one connector of the microstrip line was charged with a 50 ohm load termination, and the other connector and the sensor were connected to the VNA. With this setup, the $S_{21}$ parameter was measured and presented with respect to the calibrated reference level already stored.

**Figure 7:**
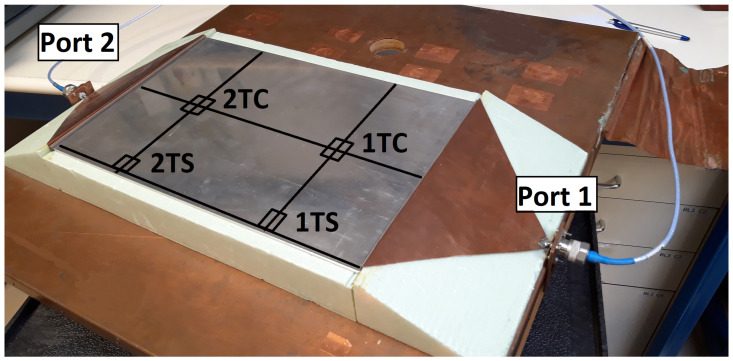
Metallic plate for calibration: imaginary lines in the direction of the current propagation (line “1T” and “2T”) and at the center (C) and end (S) of them.

In addition, measurements with the sensor were made at different points of the sample. The sample was divided into three equally spaced imaginary zones in the direction of the current propagation. Figure 7 shows the lines that separate the first imaginary zone of the samples from the second one (line “1T”) and the second one from the third one (line “2T”). As shown in the following section, the current probe was usually placed in the center of these lines (“1TC” and “2TC”) and at one end of them, along the larger side of the sample (“1TS” and “2TS”).

## 4. Results

A combination of the different laminates of MILANO listed in Section 2.1 and of the joints described in Section 2.2 were fabricated and measured. Within the adhesive joints, the following combinations were made: blue–blue, blue–orange, orange–orange, and red–red. On the other hand, within the fastener joints, the red+3–rivets–red+3 and red+3–aluminum–red+3 samples were fabricated. For reference, an aluminum plate with the same dimensions as the samples was also measured.

### 4.1. Scattering Parameter Measurements

**Figure 8:**
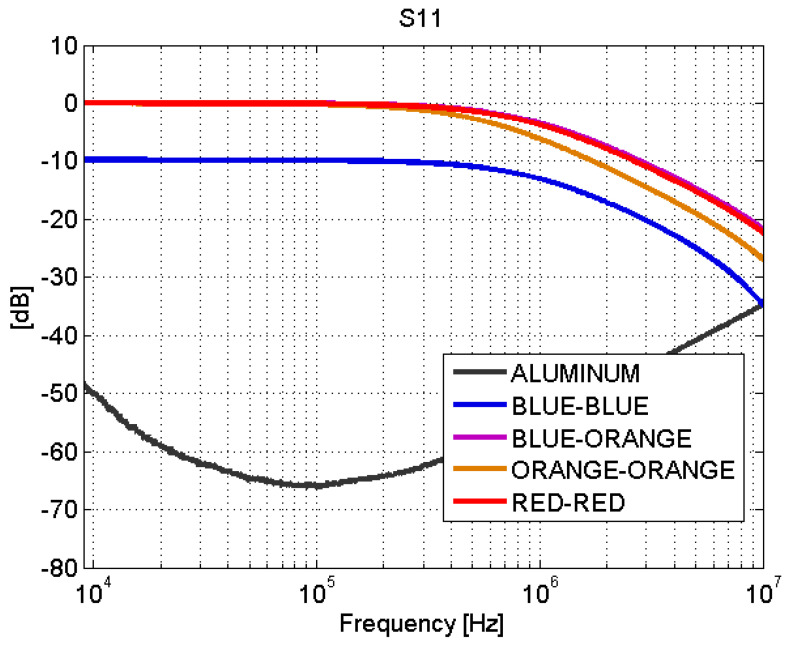
Scattering parameters test: $S_{11}$. Adhesive joints.

**Figure 9:**
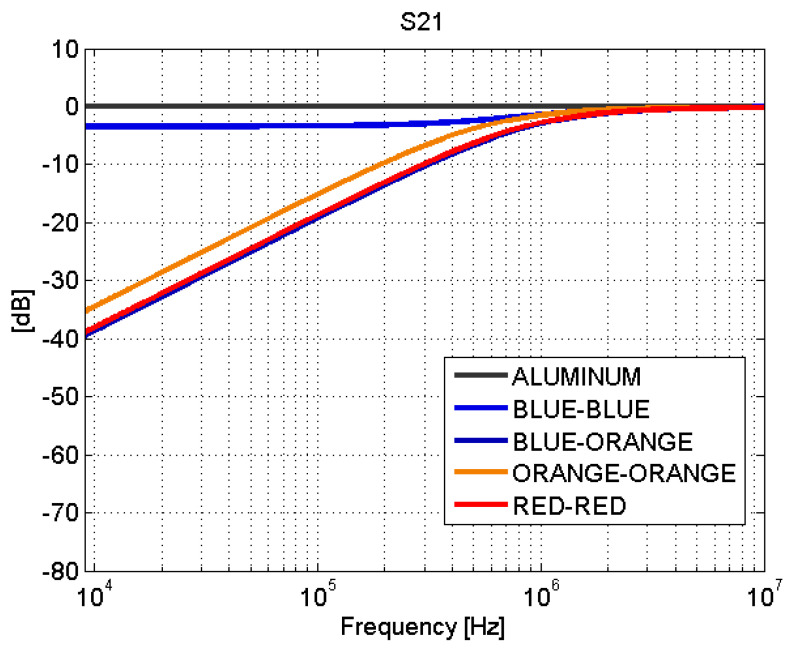
Scattering parameters test: $S_{21}$. Adhesive joints.

Depending on the type of joint, adhesive joints or fastener joints, the results obtained in the transmission line test show very different behaviors. The S-parameters for adhesive joints are shown in Figure 8 and Figure 9. For frequencies between 9 kHz and 1 MHz, a total mismatching is observed. However, from 1 MHz to 10 MHz, the samples present a better impedance adaptation and lower transmission losses. Therefore, the adhesive joints apparently present a capacitive coupling and behave similar to a high-pass filter (HPF).

**Figure 10:**
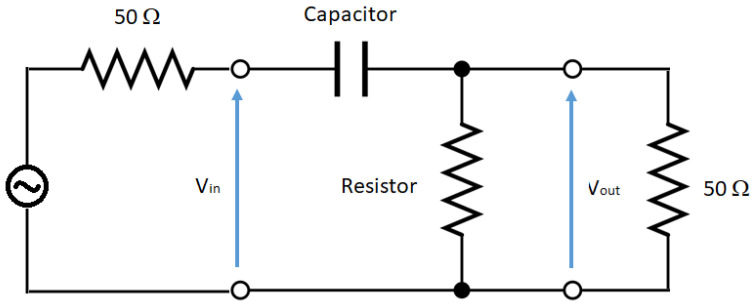
First-order high-pass filter model: this type of filter is a series combination of a capacitor and a resistor.

**Figure 11:**
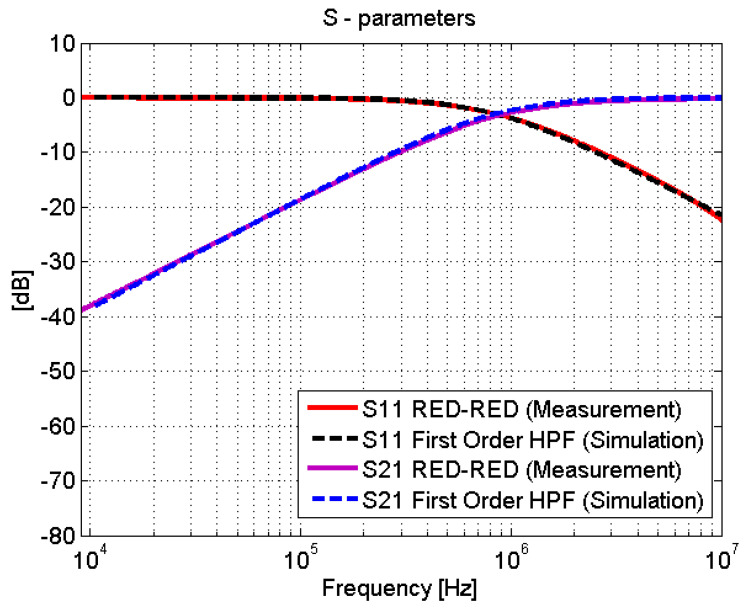
Scattering parameters test: adhesive joints vs. first-order high-pass filter (HPF).

To demonstrate this, a first-order HPF simulation was performed as shown in Figure 10. This type of filter is a series combination of a capacitor and a resistor. The voltage across the resistor is used as the output. For example, the red-red joint can be represented as a first-order HPF, following the schematic depicted in Figure 10, with a large resistance (greater than 5000 ohm) and a capacitance of 1.9 nF. Measurements and filter simulation behave identical for the studied frequencies, as shown in Figure 11. These values of capacitance and resistance are directly connected with physical effects in the joints. On the one hand, the resistor represents losses on the electric field that flows between the upper conductor of the microstrip and the ground, and its resistance value must be naturally large, provided that they are connected through a dielectric material with very low losses (the Styrodur^®^ substrate). On the other hand, the capacitance *C* that appears in the overlapping area of the two CFC parts is governed by the following formula:

(1) $$C=\epsilon \frac{A}{d}$$

where ϵ is the permittivity of the material between the CFC parts, *A* is the overlapping area, and *d* is the gap between the CFC parts. The area is 260 times 56 mm${}^{2}$ and the relative permittivity of the adhesive is 7.20 [37]; therefore, a capacitance of 1.9 nF implies a gap of 448 μm, a value that can be perfectly in agreement with the dimensions of the samples. This exercise could be reproduced for all the adhesive joints in order to electrically characterize the joint.

The blue–blue joint shows a slightly different behavior with respect to the rest of the adhesive joints. By studying the bonding process, it turns out that, before joining the CFC parts, a sanding treatment is carried out. The sanding process can leave CFC filaments without an epoxy cover that yields a good electrical contact between the sheets of the union. This explains the low transmission losses (Figure 9) in this case.

**Figure 12:**
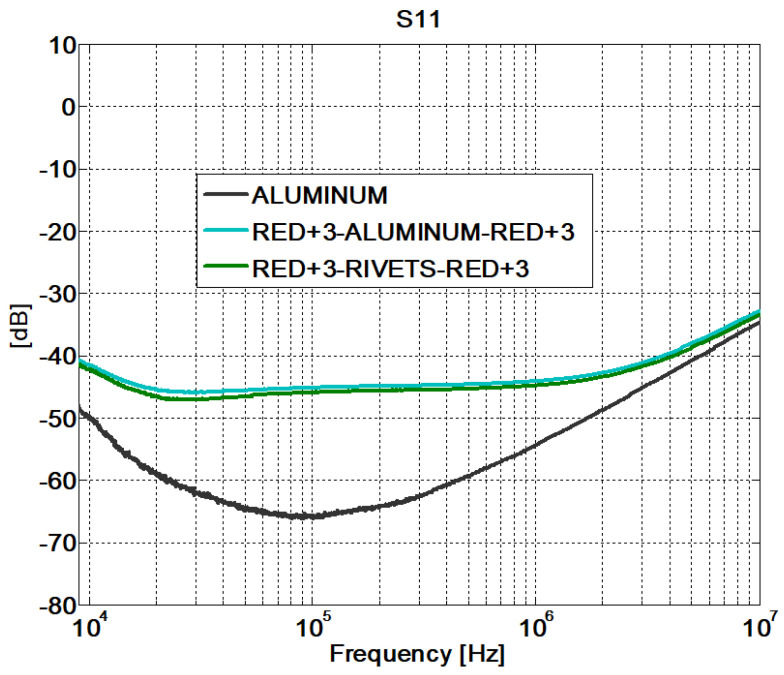
Scattering parameters test: $S_{11}$. Fastener joints.

**Figure 13:**
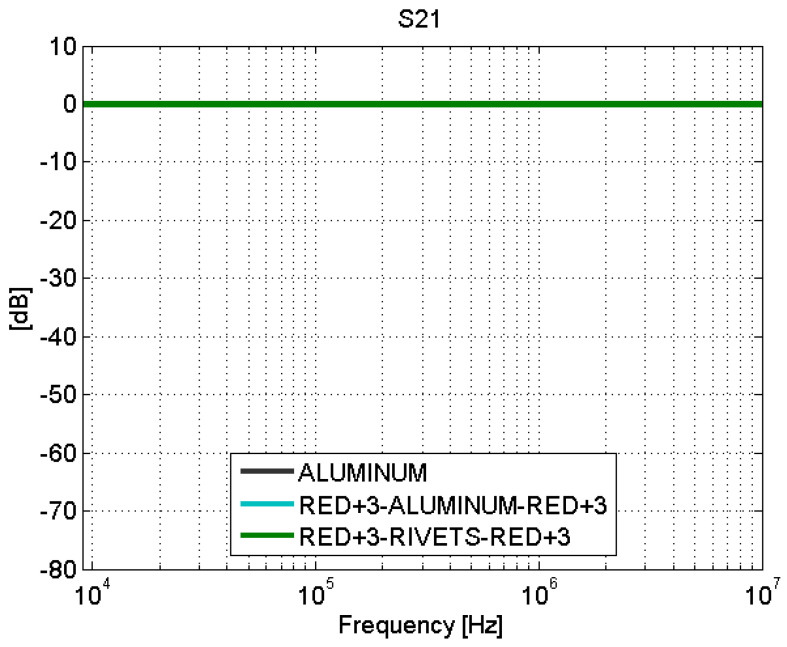
Scattering parameters test: $S_{21}$. Fastener joints.

The behavior of the fastener joints is shown in Figure 12 and Figure 13. The reflection coefficient is very low and there are no transmission losses throughout the frequency band. The behavior of these fastener joints is similar to the behavior of an aluminum sample. It can be derived that the rivets make a good electrical contact with the filaments of the CFC parts, and therefore, there is electrical continuity between them. For the aluminum sample. the matching is perfect and the losses are small, as shown in Figure 8, Figure 9, Figure 10, Figure 11, Figure 12 and Figure 13.

As mentioned before, studies of the joints in CFC structures are scarce in the literature and are limited to electric resistance methods. However, despite the materials and joints analyzed in [26] being different, it is worth noting that the S-parameters of fastener joints presented along this text are congruent with the results of bolted joints found in [26], both in magnitude and shape in frequency.

### 4.2. Surface Current Measurements

**Figure 14:**
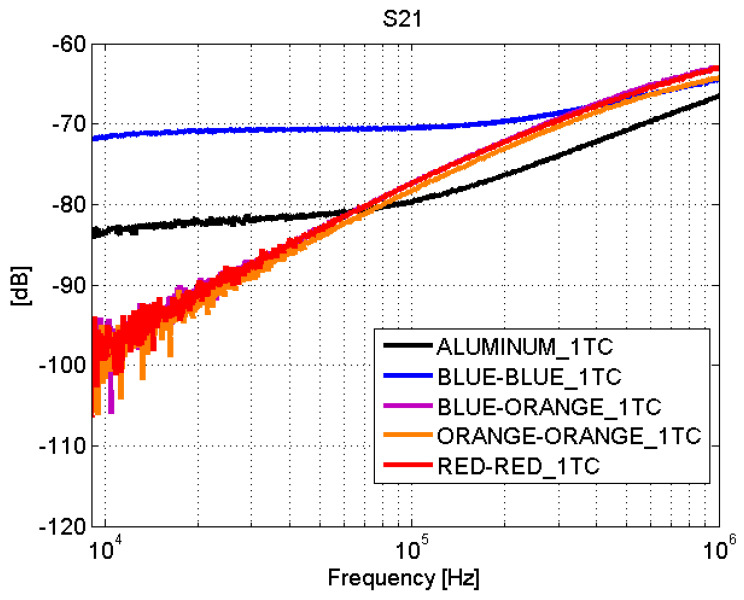
Surface current test: $S_{21}$. Adhesive joints. Line 1T.

**Figure 15:**
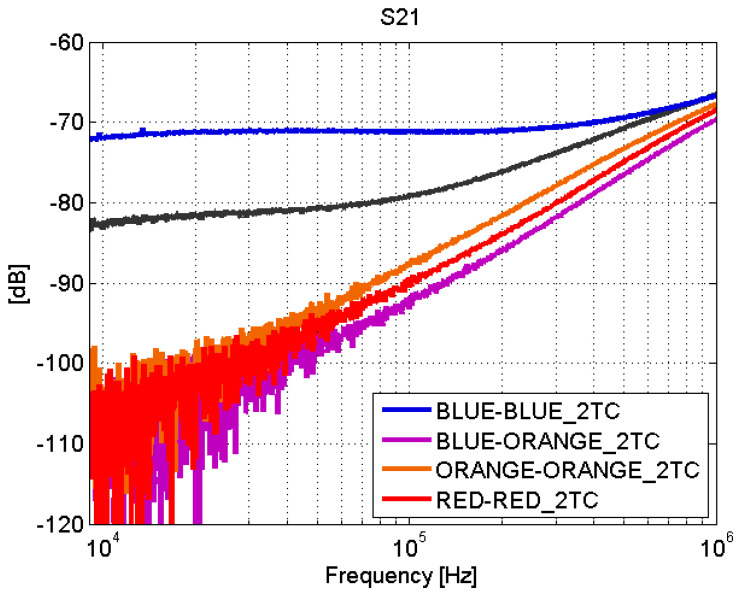
Surface current test: $S_{21}$. Adhesive joints. Line 2T.

The relative measurements of $S_{21}$ parameters with respect to the calibrated reference level are shown before the joint in Figure 14 (position “1TC”) and after the joint in Figure 15 (position “2TC”). The results show that the adhesive joint introduces losses to the surface current of 10 dB at 100 kHz and 5 dB at 1 MHz. The blue–blue joint behaves similar to the behavior of the fastener joint. An explanation of this was made in Section 4.1.

**Figure 16:**
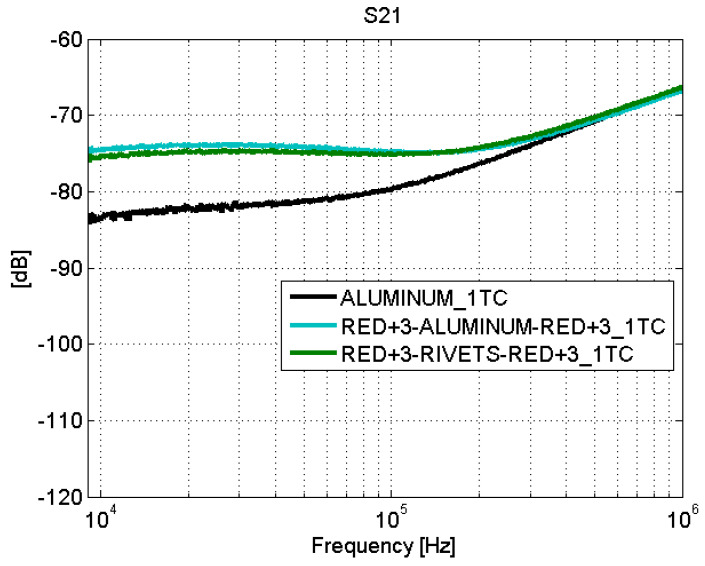
Surface current test: $S_{21}$. Fastener joints. Line 1T.

**Figure 17:**
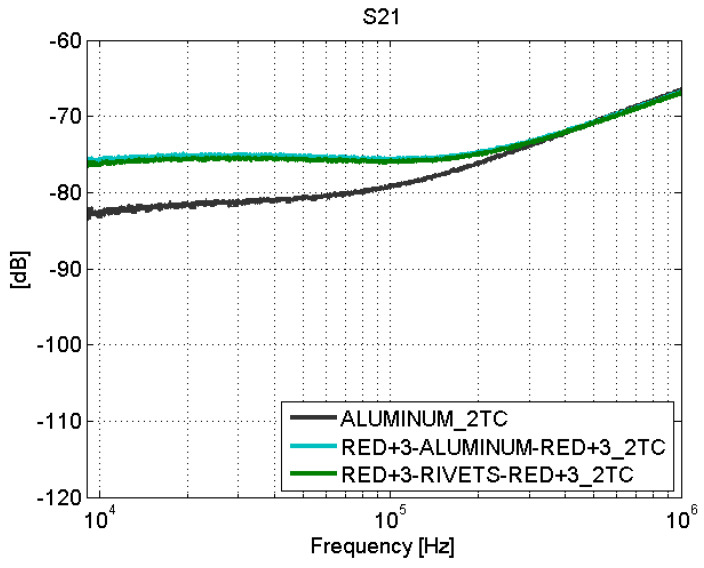
Surface current test: $S_{21}$. Fastener joints. Line 2T.

However, the fastener joints (Figure 16 and Figure 17) do not show apparent losses across the joint. It is worth noting that the comparison of the results obtained for the fastener joints samples and the aluminum sample yields that the surface current is higher in the carbon fiber samples (with fastener joint) than in the sample of aluminum. In order to assess this effect in a position different than the center of the samples, the $S_{21}$ parameter was measured with the surface current probe now also on one side (“1TS”) of a CFC sample (red+3–rivets–red+3) and the aluminum sample (see Figure 7).

**Figure 18:**
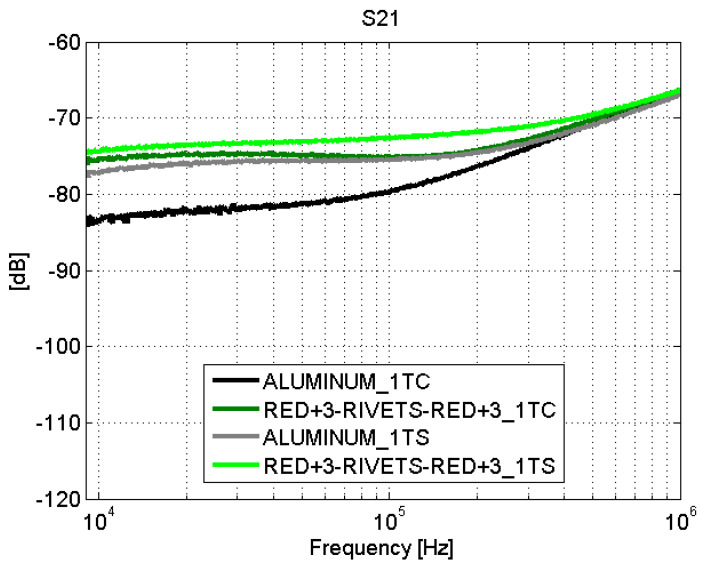
Surface current test: $S_{21}$. red+3–rivets–red+3 and aluminum. Line 1T.

**Figure 19:**
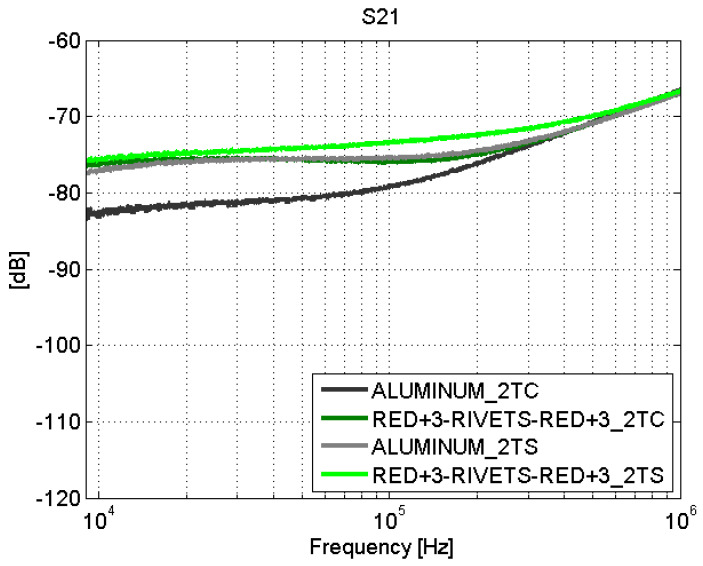
Surface current test: $S_{21}$. red+3–rivets–red+3 and aluminum. Line 2T.

The surface current seems to be more homogeneous across the CFC sample than across the aluminum sample, as shown in Figure 18 and Figure 19. An explanation for this behavior is that the distribution of conductive filaments made of carbon inside the CFC is approximately homogeneous at the macroscopic level. Then, the current flows uniformly all around the plate of CFC. On the other side, in a finite metal plate, there is an edge effect that causes preferred paths for currents to flow through them; therefore, the surface current presents different values depending on the position of the probe. Thus, the current flows easily through the edges of the metallic plate.

## 5. Conclusions

The study of the joints between different parts of an aircraft structure made of composite materials is necessary to know the electromagnetic behavior of the aerial platform. In the case of lightning effects, the study of joints is fundamental because electrical discontinuities or preferential routes can produce changes in the electromagnetic behavior of the aircraft. In this article, a measurement method was proposed and two types of joints were studied (adhesive and fastener joints).

The measurement system proposed for the characterization of the joints is a microstrip line. The chosen setup allowed the evaluation of the two types of joints in CFC samples with a different number of plies. In addition, it allowed us to make two types of tests. Measurements of S-parameters and surface current measurements were carried out in order to forecast the behavior of materials and joints against the transients generated by the direct and indirect effects of a lightning strike.

The S-parameters measurements for adhesive joints show a behavior similar to a high-pass filter. For frequencies less than 1 MHz, total mismatching is observed, whereas from 1 MHz, the samples present lower transmission losses and better impedance adaptation. This behavior can be quantified in resistance and capacitance values for all adhesive joints. The behavior of the fastener joints is similar to the behavior of an metallic sample with perfect adaptation and low transmission losses.

The surface current measurements show that the adhesive joints introduce losses between the two parts of the sample while the fastener joints have a perfect adaptation. Observing the results obtained for the fastener joints, the surface current turned out to be more homogeneous in CFC samples than in the aluminum sample. This behavior was explained based on the homogeneous distribution of carbon filaments inside the CFC parts.

An interesting outcome of this study is that faulty samples can be detected. This is what happened in the blue–blue case, a sample that presented a behavior different from the expected one. This different behavior is a consequence of a defect in the bonding process. The sanding process can leave CFC filaments without epoxy cover that yield a good electrical contact between the joined CFC parts. Therefore, the behavior of this sample is similar to the behavior of a fastener joint with less transmission losses.

The conclusions drawn on the characterization of the joints in this article allow for advancement in better understanding of the global behavior of an aircraft within an electromagnetic environment.

## Figures and Tables

**Table 1:** MILANO unmanned aerial vehicle (UAV) laminates.

Material	Stacking Sequence	Nº of Plies
red	[+45/−45/0/−45/+45/90/90/+45/−45/0/−45/+45]	12
orange	[+45/−45/+45/0/+45/90/+45/0/−45/−45/0/+45/90/+45/0/+45/−45/+45]	18
blue	[+45/−45/−45/0/+45/90/+45/0/−45/−45/0/+45/90/+45/0/−45/−45/+45]	18
	[+45/−45/0/−45/+45/90/90/+45/−45/0/−45/+45]	
red+3	[+45/−45/0/−45/+45/90/90/+45/−45/0/−45/+45]	36
	[+45/−45/0/−45/+45/90/90/+45/−45/0/−45/+45]	

## Data Availability

Not applicable.
